# Hyperbranched Polyethyleneimine–Coordinated Copper(II) Metallopolymers with Preferential Targeting to Prostate Cancer Cells

**DOI:** 10.3390/ph18081189

**Published:** 2025-08-12

**Authors:** Barbara Mavroidi, Kyriaki Marina Lyra, Stergios Pispas, Zili Sideratou, Dimitris Tsiourvas

**Affiliations:** 1 Institute of Nanoscience and Nanotechnology, National Center for Scientific Research ‘‘Demokritos”, 15310 Aghia Paraskevi, Greece; b.mavroidi@inn.demokritos.gr (B.M.); k.lyra@inn.demokritos.gr (K.M.L.); z.sideratou@inn.demokritos.gr (Z.S.); 2 Theoretical and Physical Chemistry Institute, National Hellenic Research Foundation, 48 Vassileos Constantinou Avenue, 11635 Athens, Greece; pispas@eie.gr

**Keywords:** hyperbranched polymers, metal-chelating polymers, prostate cancer cells, anticancer properties

## Abstract

**Background/Objectives**: Copper levels are significantly elevated in both the sera and tumor tissues of various cancers, including prostate cancer. It has therefore been suggested that targeting the elevated copper levels with copper chelators could lead to selective cancer treatment. Thus, several classes of low molecular weight copper-coordinating lipophilic compounds, as well as the newly developed copper complexes of appropriately functionalized polymers, are being investigated as promising novel anticancer therapeutics. Particularly, metal-containing polymers, or metallopolymers, are systematically investigated as anticancer agents or as drug delivery systems. This study aims to utilize the strong copper-chelating properties of hyperbranched polyethyleneimine (PEI) to develop PEI:Cu metallopolymers and evaluate their selectivity and anticancer properties against several prostate cancer cell lines. **Methods:** A series of PEI:Cu complexes at PEI/Cu ratios that ensure that no free copper ions are present in the solution are prepared and investigated against a human non-cancerous cell line and three prostate cancer cell lines of increasing metastatic potential. **Results:** PEI:Cu derivatives are cytotoxic against the human prostate carcinoma metastatic PC3 and DU145 cell lines, even at the lowest tested concentrations of 5 μg/mL, while against the non-cancerous HEK293 cells, all metallopolymer derivatives exhibit insignificant cytotoxicity up concentrations of 50 μg/mL. Their cytotoxic effect is associated with mitochondria membrane potential loss and ROS production increase. **Conclusions:** Hyperbranched polyethyleneimine–coordinated copper(II) metallopolymers, at low concentrations, selectively induce cytotoxicity in metastatic prostate cancer cell lines without compromising the viability of non-cancerous embryonic kidney cells.

## 1. Introduction

Copper is an essential trace element that plays a fundamental role in numerous physiological processes, including cellular respiration, angiogenesis, immune response, energy metabolism, and antioxidative defense [1]. It is a key cofactor of important enzymes that function in biological processes, including antioxidative defense and energy metabolism, and is involved in the production of neurotransmitters, the formation of connective tissue and endogenous antioxidant defense [2,3]. On the other hand, copper ion is toxic at elevated concentrations, is involved in mechanisms that promote lipid peroxidation [4], and induces the production of reactive oxygen species (ROS) [5]. Therefore, whenever copper transporters crucial for homeostasis are impaired and copper homeostasis is not maintained, copper imbalances lead to the pathogenesis of several chronic inflammatory diseases as proven by several meta-analyses and systematic reviews [1,3]. Notably, elevated copper concentrations have been observed in the sera and tumor tissues of patients with various malignancies, such as bladder and prostate cancer [6,7,8]. In the context of cancer, copper has been proposed to be involved in tumor progression through multiple mechanisms, including ROS-mediated DNA damage, promotion of angiogenesis, and the modulation of cell proliferation [9]. In prostate cancer specifically, both circulating and intratumoral copper levels are markedly increased compared to healthy controls [10,11,12]. However, it is not yet known how the increased Cu levels, along with the decreased levels of other trace elements (such as Se, Zn, and Mn), play a significant role in the initiation of prostate cancer [8,10].

It has been suggested that targeting the elevated copper levels—in both the serum and tumor—using copper chelators could provide a route for selective cancer treatment [13,14,15]. Several categories of lipophilic copper-coordinating compounds, such as hydroxyquinolines and dithiocarbamates, are currently under investigation as potential anticancer agents [16,17]. Notably, both clioquinol and disulfiram have been shown to form copper complexes capable of being transported through cell membranes and, thus, facilitating cell copper uptake, and triggering apoptosis [14,18,19,20,21]. To further explore the therapeutic potential of disulfiram, a translational pilot trial (Clinicaltrials.gov, identifier: NCT01118741) was conducted [22]. As evidenced by a number of recent reviews, copper-based complexes and nano-formulations are currently intensively explored in cancer research [23,24], and, in particular, the role of copper complexes and ionophores as prostate cancer therapeutics has been explored, focusing on the underlying mechanisms and their therapeutic potential [25]. Although the precise mechanisms underlying the selective toxicity of these agents towards cancer cells remain unclear, it is of interest to note that studies on ^64^CuCl_2_, a promising radionuclide studied as a theranostic agent for cancer imaging and diagnosis, have shown promising results. In particular, ^64^CuCl_2_ induces substantial cytotoxic effects in prostate cancer cells while exerting minimal impact on healthy tissues. This was attributed to inadequate mechanisms for repairing DNA damage in prostate cancer cells, in contrast to effective repair processes in non-tumor cells that are able to efficiently repair the biological effects induced by the radionuclide [26]. Its effectiveness was not only observed in 2D cellular cultures, but also in spheroids of castration-resistant (22RV1 and DU145) and hormone-naïve (LNCaP) cell lines, reducing their growth and reproductive ability [27]. More importantly, it was found that prostate cancer cells exhibited higher ^64^Cu uptake and significant nuclear internalization compared to non-tumoral prostate cells [26,27].

The promising results of copper(II) low-molecular weight complexes as anticancer medications triggered the development and study of polymeric copper complexes. In general, metal-containing polymers, also referred to as metallopolymers, are a subgroup of polymers that are currently systematically investigated as anticancer agents or as drug delivery systems [28,29]. Within the emerging class of metallopolymers, particular attention has been given to metallodendrimers, metallodendrons, and metallohyperbranched polymers, i.e., dendritic macromolecules in which metal ions are incorporated at various positions throughout their dendritic structure. The metal centers (either main group metals, transition metals, or lanthanides) are attached to the repeating units either covalently through an organic linker, or non-covalently through metal chelation [30]. Covalent attachment results in irreversible metal binding, whereas chelation results in a reversible coordination between metal ions and donor atoms within the polymer’s repeating units [31]. This dynamic and reversible binding is advantageous in the sense that it allows the metal ion release within a cell, if the appropriate conditions are met [30,31]. As a result, there is growing interest in developing hyperbranched polymeric metal chelates due to the reversible nature of coordination bonding, as well as due to their low cost and availability [31,32,33]. An additional advantage is their large number of end-groups that, after further functionalization, can lead to novel properties with biochemical and biological significance [34].

**Scheme 1:**
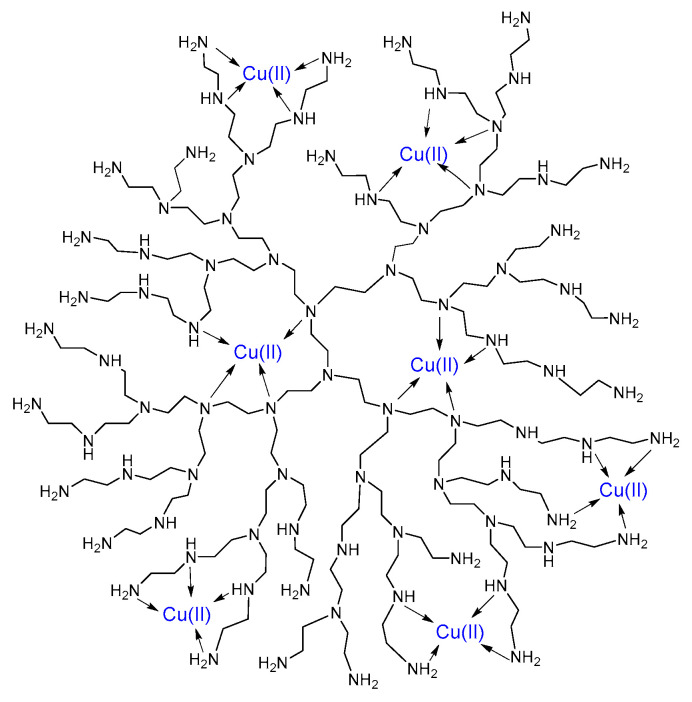
Schematic representation of possible structures of polyethyleneimine-coordinated copper(II) metallopolymer, where Cu(II) coordinates to primary, secondary, and tertiary nitrogens of PEI.

Hyperbranched polyethyleneimine (PEI) is the most commonly employed polymer for the efficient chelation of metal ions [31]. PEI is a scaffold that is utilized not only for the development of drug and gene delivery systems [35,36], but also exhibits antimicrobial properties against bacteria [37,38,39]. It has a chemical structure consisting of the ethylenediamine-type branches (Scheme 1) of strong coordination ability for a wide range of metal ions in aqueous solutions. In particular, PEI forms blue water-soluble complexes with copper(II) ions (PEI:Cu) that are stable over a wide pH-range (4 < pH < 9) [40,41,42,43,44,45,46,47]. The reported equilibrium constant is approximately 10^16^ [40,44,46], which is larger than the corresponding formation constants of ethylenediamine, and close to that of diethylenetriamine [46,48]. This is attributed to the large local concentration of the ligands within the hyperbranched structure and to the resulting efficient coordination of the metal ions by two ethyleneimine-repeating units [48]. In total agreement with the above is the fact that hyperbranched polyethyleneimine compared to its linear polyethyleneimine counterpart leads to a more favorable complexation. In addition, the molecular weight and microstructure of PEI have minimal effect on the chelating capacity, which is in sharp contrast with low-molecular-weight tetramines—whether linear or branched—which exhibit significant variations in their metal-binding efficiency [46].

Previous studies on PEI:Cu-based polymeric micelles or nanoparticles revealed that they are ideal Cu(II) ion carriers. PEI-based synthesized nanoparticles can coordinate copper ions and enable their transport across eukaryotic cell membranes, which could then be released intracellularly and be accessible for biological processes; their reduced toxicity compared to free Cu ions indicated that Cu was effectively shielded by the NPs [49,50,51]. It was also found that the supplementation of the medium with Cu-complexed nanocarriers resulted in elevated intracellular copper levels and enhanced specificity in copper transport [50]. Similarly, ^64^Cu-labeled polyethyleneimine was studied both for cell trafficking and tumor imaging, while PEGylation further reduced its toxicity and improved tumor imaging ability and xenograft tumor visualization [52]. These findings can be rationalized according to Tweedy’s chelation hypothesis [53] that the metal chelation considerably reduces the metal ions polarity, thus facilitating their transmembrane transport through the lipid bilayer.

In the present study, we prepared a number of copper(II) coordination complexes with PEI including Cu:primary amino groups of PEI molar ratios ranging from 1:32 to 1:4, i.e., at considerably lower molar ratios than those previously studied, so as to ensure the full chelation of Cu(II) ions. In this way, we can solely survey the effect of PEI:Cu complexes against a human non-cancerous cell line (HEK293) and three prostate cancer cell lines of increasing metastatic potential (LNCaP, DU145, and PC3). Our aim was to examine the effect of varying PEI:Cu molar ratios on the physicochemical properties of the metallopolymers and their in vitro effect on cancerous vs. non-cancerous cell lines.

## 2. Results

### 2.1. PEI–Cu(II) Complexes Formation

**Figure 1:**
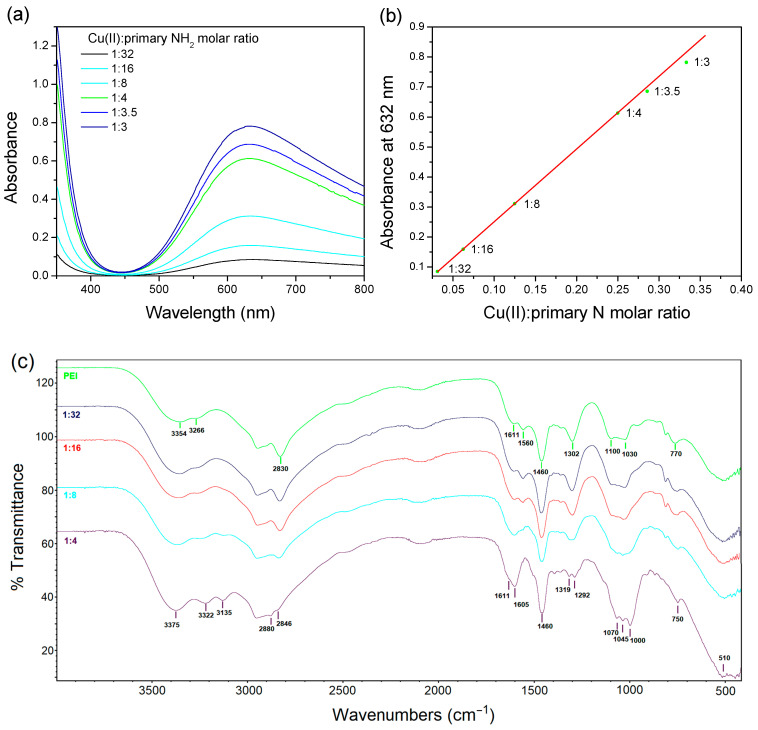
Spectra of PEI–Cu(II) complexes of various Cu(II):N molar ratios: (**a**) UV–Vis spectra in phosphate buffer (pH 7.4, polymer concentration 2 mg/mL, optical path length 1 mm) and (**b**) plot of absorbance at 632 nm; (**c**) FTIR spectra of dried PEI–Cu(II) complexes.

Copper ions complexation to PEI, initially published by Thiele et al. [40], has been thoroughly studied ever since by many research groups [41,42,43,44,45,46,47] that verified that branched polyethyleneimines of different sources and of a variety of molecular weights and degrees of branching always form blue-colored water soluble complexes with copper(II) ions, with an absorbance maximum at 632–635 nm [40,44,48,49,54]. Due to the strong basic nature of PEI, all complexation experiments were conducted in phosphate buffer (PB, 100 mM, pH 7.4) to maintain a neutral pH at the PEI concentrations used (up to 200 µg/mL). To avoid the presence of unbound Cu(II) ions in solution, it was essential to determine the maximum Cu:primary amine molar ratio (Cu:N) that ensures complete copper complexation. Unlike previous studies that employed Cu:PEI molar ratios corresponding to the maximum stoichiometric quantity of Cu, we intentionally employed significantly lower ratios to guarantee the full chelation of Cu(II) ions. To this end, we monitored the absorbance maxima at 362 nm of various PEI:Cu complexes in PB as a function of added Cu(II) ions using UV–Vis spectroscopy, taking advantage that both PEI solutions and free Cu(II) solutions have negligible absorption at this wavelength. The absorbance is expected to increase linearly with an increasing Cu:N ratio in the case of the full complexation of the anion, while deviation from the straight line signifies the presence of free, uncoordinated Cu(II) ions [55,56]. As shown in Figure 1a, the UV–Vis spectra of all PEI:Cu complexes have maximum absorbance at 632 nm, while the absorption profile as a function of the Cu:N molar ratio (Figure 1b) revealed a deviation from linearity beyond a 1:4 molar ratio, indicating that this is the upper limit for complete Cu(II) binding under the conditions employed. Consequently, a Cu:N molar ratio of 1:4 was selected as the highest ratio for subsequent experiments. It should be noted that, in all previous publications on PEI-Cu complexes, the Cu:total number of PEI amino groups employed was significantly higher, equal to four or even five (noted in the literature as CuN4 and CuN5), which was based on the theoretically expected formation of four- or five-coordination complexes [40,41,42,46]. Our above results led us to employ significantly lower molar ratios ensuring complete copper ions coordination. The observed maximum of absorbance at 632 nm in line with the literature indicates that four N atoms are coordinating one Cu(II) ion with a planar array of the four Cu–N bonds [46,47,55,57]. Since PEI contains primary, secondary, and tertiary nitrogens, it can be considered as a macromolecular analog of both linear and branched oligoamines which are known to form strong, planar, Cu(II) complexes [46,54,58]. It is, therefore, possible that, in this case also, Cu is coordinated to either primary, secondary, or tertiary nitrogens of PEI, as tentative illustrated in Scheme 1.

The effect of the complexation was also followed by FTIR spectroscopy (Figure 1c), as it is known that the complexation of PEI amino groups with copper ions is expected to result in shifts of the C-N vibration modes to lower frequencies [44]. Indeed, the bands in the 1100–1000 cm^−1^ region related to the C-N stretching modes are shifted to the right and also increase in intensity. It has also been suggested that the formation of N-Cu coordination bonds increases the electron demand of nitrogen atoms, and consequently, the polarity of the N-H bond, which would result in shifts of the N-H vibrations and in an increase in their intensity [59]. As shown in Figure 1c, this is also evident in our spectra since, upon the increase in the Cu:N molar ratio, the vibration modes in the FTIR spectra of dried polyethyleneimine complexes undergo changes especially in the stretching, deformation, and rocking modes of N-H groups. Specifically, as expected [44,59,60,61], i76n the FTIR spectra of the complexes with high Cu content (Cu:N 1:8 and 1:4), we observe changes both in the position as well as in the intensity of the bands in the 3500–3000 cm^−1^ region, corresponding to the stretching N-H modes, in the 1600–1500 cm^−1^ region, corresponding to the deformation frequencies of NH and NH_2_ groups, as well as for the NH_2_ rocking vibration in the 800–750 cm^−1^ region. Interestingly, the C-H stretching band at 2830 cm^−1^, which is assigned to the α-CH_2_ groups next to N, also diminishes in intensity upon the complexation of the adjacent nitrogen atom, in full accordance with previous similar studies on N-alkylated polyethyleneimines–copper complexes [62,63]. Finally, the new band at 510 cm^−1^ has been assigned to the newly formed Cu-N stretching mode [60,61].

In order to study the variations in size and size distributions as well as in the electrophoretic mobility of the metallopolymers as a function of the Cu:N molar ratio, we resorted to the use of hyperbranched PEI 750 kDa and its Cu complexes, since PEI 25 kDa, due to its low molecular weight, was outside the useful working range of both dynamic light scattering (DLS) and zeta-potential instruments. This can be justified due to the fact that both hyperbranched polyethyleneimines have the same branching degrees and the same primary–secondary–tertiary ratios as determined by the inverse gated ^13^C NMR analysis (see experimental section). Zeta-potential measurements of PEI 750 kDa and the corresponding PEI–Cu(II) solutions (5 mg/mL, 25 °C) revealed a very small variation of polymer electrokinetic potential upon the complexation with Cu ions (Figure S1a), which was rather unexpected since the presence of the positively charged Cu(II) ions in the polymeric matrix would be anticipated to substantially increase the polymer charge. In particular, the zeta-potential values of complexes with Cu:N ratios from 1:32 up 1:8, point to a minor, although not statistically significant, reduction in their electrokinetic potential compared to the parent polymer. This overall behavior can be tentatively ascribed to the neutralization of the positively charged nitrogen atoms (at neutral pH, a large number of nitrogen atoms of PEI are positively charged) upon their complexation with Cu, which leads to an overall similar net charge density of the polymeric matrix. Alternatively, this could be the result of changes in the overall conformation of the macromolecule upon the complexation with copper ions that could, in turn, affect its electrokinetic mobility.

Dynamic light scattering experiments of PEI 750 kDa and the corresponding PEI:Cu(II) solutions at the same concentration as above (5 mg/mL) revealed a significant change in polymer size and size distributions (Figure S1b). The uncomplexed PEI has a broad size distribution centered at 50 nm, accompanied by a minor shoulder at ~ 10 nm, while the DLS of PEI:Cu complexes having Cu:N molar ratios of 1:32 and 1:16 reveal the presence of narrower size distributions centered at 10–15 nm, which increased in intensity upon the increase in the Cu:N ratio at the expense of the peak at ~50 nm. It is of interest to note that this is also accompanied by a minor decrease in zeta-potential (cf. Figure S1a). Both observations can be tentatively attributed to the coordinating Cu(II) ions that compel ethyleneimine branches to approach each other (cf. Scheme 1), affording more compact nanoparticles, coupled with the neutralization of the positively charged nitrogen atoms upon their complexation with Cu(II) at these molar ratios.

**Figure 2:**
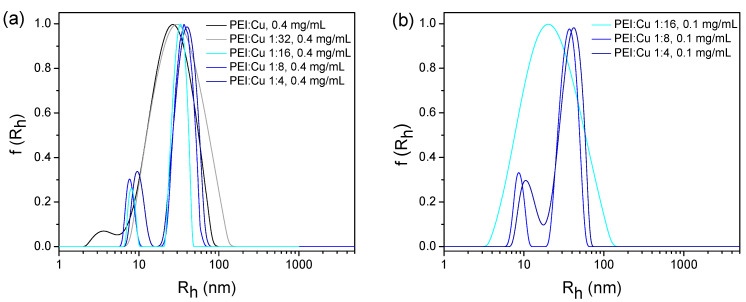
Intensity weighted hydrodynamic radii distributions of PEI of 750KDa molecular weight (PEI750) and of the corresponding PEI750:Cu solutions (pH 7.4) at two different polymer concentrations: (**a**) 0.4 mg/mL; (**b**) 0.1 mg/mL. Data shown are the mean of at least three dynamic light scattering measurements.

It is evident that conformation changes of the polymeric complexes as a function of Cu:N ratios are taking place, which also led us to explore in more detail their behavior at lower polymer concentrations, i.e., at concentrations close to those used in cell culture experiments (see below). Thus, at 0.4 mg/mL, the parent polymer as well as the polymer with a Cu:N ratio of 1:32 have broad size distributions centered at ~30 nm. However, at a Cu:N ratio of 1:16, two distinct narrow size distributions begin to form centered around ~9 and 35 nm, while at higher Cu:N ratios up to 1:4, the two distributions are fully resolved (Figure 2a). At even lower concentrations (0.1 mg/mL; Figure 2b), the same behavior is observed: although, in this case, the scattering signal is very low in intensity, it is clear that, up to a Cu:N ratio of 1:16, broad distributions are observed, whereas, at Cu:N ratios of 1:8 and 1:4, two narrow size distributions are evident at ~10 and 35 nm. In summary, a clear change in the polymer size and conformation is observed upon increasing Cu(II) content, resulting in compact nanoparticles of a few nanometers and with a very narrow size distribution. This is tentatively attributed to the formation of a significant number of Cu:N coordination bonds that bring together neighboring ethyleneimine branches (cf. Scheme 1).

### 2.2. In Vitro Cell Studies of PEI:Cu Metallopolymers Against Cancerous and Non-Cancerous Mammalian Cell Lines

**Figure 3:**
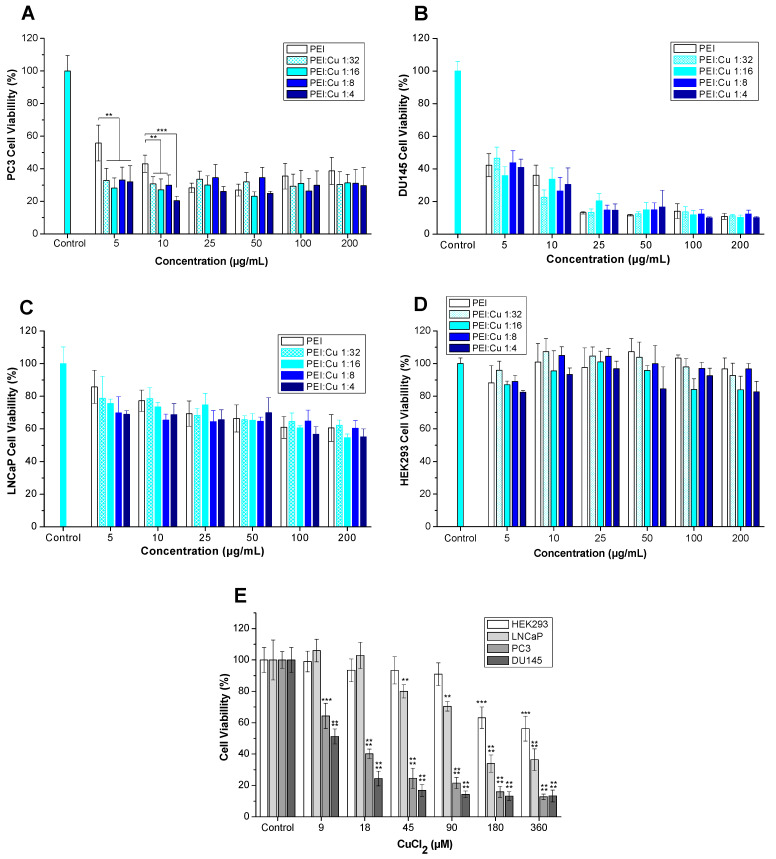
Toxicities of PEI as well as of PEI:Cu complexes on PC3 (**A**), DU145 (**B**), LNCaP (**C**), and HEK293 (**D**) cells lines following incubation at various concentrations for 3 h. After a 3 h incubation period, the wells were thoroughly washed and further incubated in complete media for the next 24 h. The mitochondrial redox function (translated as cell viability) was then quantified by the MTT assay. The cytotoxicity of CuCl_2_ at concentrations that correspond to the nominal copper ion concentration of the PEI:Cu 1:4 derivative was also assayed (**E**). Data are presented as mean ± SD from six replicates collected across at least three independent experiments, and analyzed relatively to the respective untreated cells (control) using the Student’s *t*-test, following the assignment: ** *p* < 0.01, *** *p* < 0.001, **** *p* < 0.0001, while no annotation implies no statistical significance, *p* > 0.05.

The cytotoxicity of PEI:Cu metallopolymers were investigated against the human prostate androgen-sensitive LNCaP and the androgen-insensitive cell lines with moderate, DU145, and high, PC3, metastatic capacity, as well as the non-cancerous human embryonic kidney HEK293 cell line. Cells were treated with PEI:Cu derivatives having Cu:N ratios of 1:4, 1:8, 1:16, or 1:32, at various concentrations (5–200 μg/mL) for an incubation time of 3 h. Subsequently, cell viability was measured by the MTT assay after further incubation for 24 h. For comparison, the PEI cytotoxicity was also assessed under identical conditions in the same concentration range, as well as free Cu(II) ions toxicity in the concentration range of 10–370 μM that corresponds to the Cu(II) content of the PEI:Cu 1:4 derivative at its concentration range employed (i.e., 5–200 μg/mL). As shown in Figure 3A,B, PEI as well as all PEI:Cu derivatives are cytotoxic against the human prostate carcinoma PC3 and DU145 cell lines, not only at relatively high concentrations but even at the lowest tested concentrations of 5 and 10 μg/mL. It is of interest to note that, at these low concentrations, PEI:Cu complexes were more toxic than PEI against PC3 cells, as the cell viabilities registered for all complexes were approximately 30% compared to the corresponding value registered for PEI (55%, *p* < 0.01), suggesting different biochemical paths between PEI and PEI:Cu complexes. On the other hand, the metallopolymers exhibit medium cytotoxicity against the androgen-sensitive LNCaP cells (Figure 3C) that are known to have low metastatic potential. Most importantly, against the non-cancerous HEK293 cells, all metallopolymer derivatives exhibit insignificant cytotoxicity concentrations of up to 50 μg/mL (Figure 3D), while even at the highest concentrations tested (200 μg/mL) the cytotoxic activity was very low (cell viabilities > 80%). At all concentrations tested the PEI:Cu 1:4 polymer is slightly less toxic against HEK293 cells compared to other polymer complexes or parent PEI (Figure 3D), although the observed difference is not statistically significant. Free Cu(II) ions, at the corresponding concentration range as in PEI:Cu 1:4, show significant toxicity against all tested cell lines (Figure 3E), although again, they are more toxic against PC3 and DU145, and less toxic against LNCaP and HEK293 cells.

**Figure 4:**
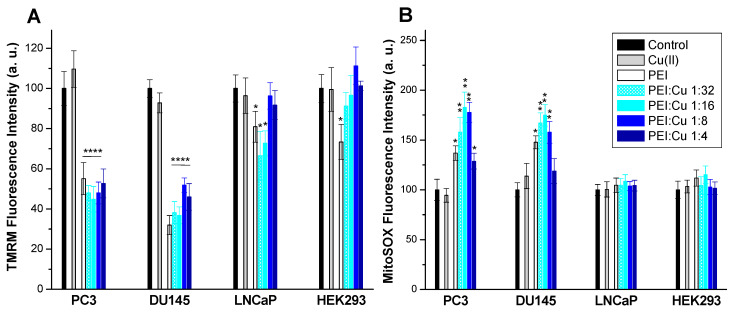
TMRM (**A**) and MitoSOX (**B**) fluorescence intensities registered after the treatment of PC3, DU145, LNCaP, and HEK293 cells lines with 5 μg/mL solutions of PEI or PEI:Cu complexes for 3 h. Data are expressed as mean ± SD from six replicates, collected across at least three independent experiments and analyzed for each cell line relatively to the respective untreated cells (control) using the Student’s *t*-test, following the assignment: * *p* < 0.05, ** *p* < 0.01, **** *p* < 0.0001; no annotation denotes no statistical significance, *p* > 0.05.

To examine whether the observed redox change in cell mitochondria as deduced by the MTT assay is also associated with mitochondria membrane potential loss, HEK293, LNCaP, PC3, and DU145 cells were treated with 5 μg/mL of either PEI or PEI:Cu complexes and then stained with TMRMs as described in the experimental section (Figure 4A). For comparison, all cells were also treated with free Cu(II) ions at a concentration of 9 μΜ that corresponds to the nominal copper concentration of a 5 μg/mL solution of the PEI:Cu 1:4 complex. For free Cu(II) ions, it is evident that all cell lines showed mitochondrial TMRM staining levels equal to those of untreated cells, so that the significant Cu ion toxicity at 9 μM observed against PC3 and DU145 (cf. Figure 3E) is not associated with the loss of mitochondrial membrane potential. On the contrary, the toxicities of PEI:Cu complexes against all cell lines are in agreement with mitochondrial membrane potential loss. HEK293 cells treated with PEI:Cu complexes showed mitochondrial TMRM staining similar to that of untreated cells, in line with MTT data that indicate minor cytotoxicities for all complexes. In contrast, all complexes significantly reduce the TMRM fluorescence of PC3 and DU145 cells, suggesting that the registered cell cytotoxicity of these compounds is accompanied with mitochondrial membrane potential loss. LNCaP cells show a medium TMRM fluorescence loss upon treatment with PEI and PEI:Cu compounds with low molar ratios (1:32 and 1:16), which is also in line with the MTT results. Finally, no significant difference between the polymeric complexes with different PEI:Cu molar ratios can be substantiated, but it is clear that, at least for the LNCaP and HEK293 cell lines, the PEI:Cu complexes with Cu:N molar ratios of 1:8 and 1:4 are definitely affecting the membrane potential less than PEI and complexes with Cu:N molar ratios of 1:32 and 1:16. Lastly, parent PEI, although it is not found to be significantly cytotoxic at this concentration, does cause a measurable reduction in mitochondrial membrane potential, suggesting a different mode of interaction with the mitochondria of PEI compared to PEI:Cu complexes in this non-cancerous cell line. Overall, the among the PEI:Cu complexes, the ones with 1:8 and 1:4 Cu:N molar ratios are the most promising as they do not negatively affect non-cancerous cells.

Given that Cu(II) is a redox-active metal promoting the production of reactive oxygen species (ROS), cells treated with 5 μg/mL of PEI or PEI:Cu complexes, or with free Cu(II) ions at a concentration of 9 μΜ, were stained with MitoSOX Red to detect mitochondrial ROS, especially superoxide anions that are the predominant ROS in mitochondria, as detailed in the experimental section (Figure 4B). Control cells as well as cells treated with uncomplexed (free) Cu(II) ions displayed low MitoSOX Red signals, an indication that they have no significant effect on superoxide anion production, not even against the PC3 and DU145 cells for which free copper ions are considerably cytotoxic (~50% cell viability, cf. Figure 3E) at this concentration. Similarly, all polymeric copper complexes have no effect on superoxide anions production in HEK293 and LNCaP cells, in line with the observed non-toxicity of the polymers against these cell lines. On the other hand, as in TMRM experiments, the incubation of PC3 and DU145 cells with PEI:Cu polymers significantly increased the ROS production, even at this low concentration, while it is of interest to note that this increase is more substantial compared to PEI.

**Figure 5:**
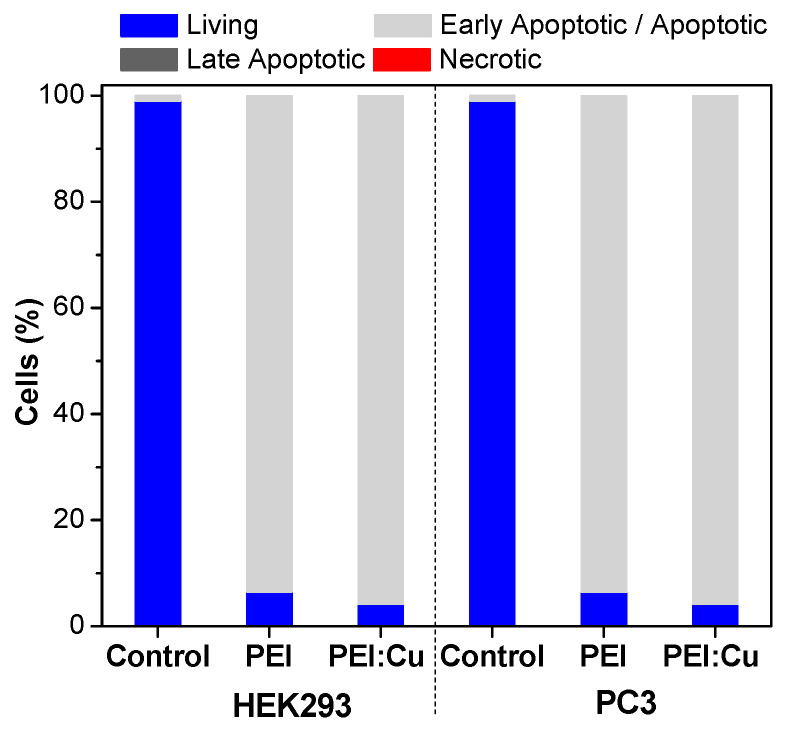
Quantification of living and early apoptotic/apoptotic percentages of HEK293 and PC3 cells treated with either PEI or PEI:Cu 1:4 derivative (5 μg/mL, 3 h), as determined following Annexin V-FITC/7-AAD staining. Cells were stained according to the conventional Annexin V/&AAD protocol as described in the experimental section. No late apoptotic or necrotic cells were observed under the employed conditions. Experiments were performed as independent duplicates.

Further insight on the anticancer activity of PEI:Cu complexes against PC3 cells vs. their cytocompatibility against HEK293 cells was attempted by the quantification of living, apoptotic, and necrotic percentage of these two cell lines. Cells were treated with 5 μg/mL of either PEI or PEI:Cu 1:4 for 3 h, and, following a further 24 h incubation period, were stained using the Annexin V-FITC/7-AAD double-staining assay, and the respective fluorescence signals were recorded by flow cytometry, as detailed in the experimental section. The assay is based on the well-established labeling of apoptotic cells by Annexin V-FITC, whereas cells with compromised membranes are only stained by 7-AAD [64]. As shown in Figure 5 (and in Figures S2 and S3), the treatment of HEK293 proves that both tested compounds (PEI and PEI:Cu 1:4) have no effect on cell proliferation. On the contrary, both PEI and a PEI:Cu ratio of 1:4—even at this low concentration—result in PC3 cells that are Annexin V—but not 7-AAD—positive. This is an indication that the phosphatidylserine residues are translocated to the outside of the cell without, however, a loss of membrane integrity, denoting that at this low concentration, cells are committed to apoptotic death in a slow and rather controlled process [65].

## 3. Discussion

The effect of polyethyleneimine copper complexes was investigated against three prostate cancer cell lines. The standard prostate cancer cell lines commonly used in therapeutic research, i.e., the androgen-sensitive with low metastatic potential LNCaP cell line, and the androgen-insensitive cell lines with moderate, DU145, and high, PC3, metastatic capacity, were employed. For the formation of a number of PEI-copper metallopolymers in phosphate buffer (PB, pH = 7.4, 100 mM), various Cu:nitrogen molar ratios were initially studied, specifically the molar ratio of Cu ions to primary amino groups of PEI (Cu:N), to establish the maximum possible ratio that fully secures the complete binding of Cu(II) ions, and avoid any effects in cell experiments that could result from uncomplexed copper ions. Thus, in contrast to previous studies that typically employed Cu:PEI total amino groups ratios up to 4 or even 5 [40,41,42,46], in this study, PEI was complexed with Cu at considerably lower molar ratios. We established that employing a Cu:N molar ratio up and equal to 1:4, the complete coordination of Cu(II) by the nitrogen atoms of PEI is fully ensured. Physicochemical analysis revealed that, upon complexation with increasing Cu:N molar ratios (from 1:32 to 1:4), the zeta-potential values (i.e., the electrophoretic mobility) remain essentially the same to that of uncomplexed PEI, whereas the mean size with increasing PEI:Cu molar ratio is reduced to a narrow size distribution in contrast to the rather broad size distribution of parent PEI. This result is in line with previous publications where the analysis of the binding isotherm of the Cu(II)-PEI complexation indicated a ligand-induced conformational change in the polymer upon the uptake of copper ions [45]. It was advocated that, upon complexation, the mobility and conformational freedom of polymer branches is restrained, whereas such ligand-induced conformational changes were not observed in the case of crosslinked PEI because, in this case, the polymer chains are locked due to crosslinking [45]. A similar case of conformational change induced by copper chelation was also reported for the albumin–copper complex [66].

Our in vitro cell viability studies provide evidence that the DU145 and PC3 prostate cancer cell lines, known for moderate and high metastatic potential, respectively, are very sensitive to very low concentrations of PEI:Cu derivatives, in contrast to the low metastatic LNCaP cells. More interestingly, the non-cancerous HEK293 cells are not affected even at high concentrations of PEI:Cu metallopolymers. The observed toxicity against DU145 and PC3 cells is accompanied with mitochondrial membrane potential loss and an increase in mitochondrial ROS—especially superoxide ions—production. In this context, it is of interest to note that their cytotoxic effect is similar, but not identical, to PEI cytotoxicity. It has been proposed that PEI toxicity is the result of cell membrane penetration/disruption, that it then induces mitochondrial membrane potential loss and finally mitochondrial-mediated cell apoptosis [67]. The PEI-Cu complexes also affect mitochondria and result in apoptotic cell death, but there is no evidence of membrane integrity loss—at least at the low concentrations tested—and, more importantly, show a specificity towards certain cell lines suggesting a different cytotoxicity mechanism than PEI.

The fact that the prostate cancer cell lines with metastatic potential are more sensitive to PEI:Cu derivatives compared to the non-cancerous HEK293 cells, can be, initially, attributed to the already well-known sensitivity of these cell lines to free Cu(II) ions [9,14,26,27,50]. Although copper is an essential metal, it is toxic at elevated concentrations and, and thus, the upper limit for Cu in drinking water is set by the United States Environmental Protection Agency to 1.3 mg/L, which is equivalent to a concentration of approximately 50 μM Cu(II) ions. It is also known that the brain and liver are the main organs susceptible to Cu toxicity. In this connection, it is reported that, in the in vitro experiments, the exposure of brain human cell lines to Cu(II) ions with a concentration above 50 μM has detrimental effects, leading to cell toxicity and increased ROS formation [68]. Our experiments corroborate previous reports since, as shown in Figure 3D, the IC_50_ of CuCl_2_ is >360 μM for HEK293 cells, while for DU145 and PC3 cells, the IC_50_ values are reduced to ca. 10 μM. This large difference in toxicity between cancerous cells vs. non-cancerous cells is in line with previous publications demonstrating that copper-64 chloride, which is studied as a suitable molecule for both high-resolution positron emission tomography imaging and for therapeutic purposes, is cytotoxic against either monolayer-cultured or the multicellular spheroids of prostate cancer cells, while exhibiting minimal cytotoxicity against non-tumoral cells. In addition, these cells exhibited higher Cu-64 uptake compared to healthy cells, and also showed significant copper nuclear internalization [26,27]. Furthermore, intracellular copper internalization enhancement was also reported for copper complexing agents, such as copper-binding serum amino acids [69], or copper-coordinating lipophilic compounds (e.g., clioquinol, disulfiram, or bis-thiosemicarbazones) [14,16,17,18,19,20,21,22], that are promising anticancer therapeutics as they are reported to selectively target prostate cancer cells. It has been suggested that copper complexes result in increased intracellular copper when administered to cells, while in one case, the dissociation of the coordinated copper was reported, which led to the increase in bioavailable copper, exposing cancerous cells to ionophoric copper sensitivity [70].

On similar grounds, a number of Cu(II)-complexing metallodendrimers have also been developed and their anticancer potential evaluated, taking into consideration their particular structural characteristics [71]. A methylpiperazine end-group functionalized poly(amidoamine) dendrimer complexed with Cu(II) ions exhibited cytotoxic effects on tumor cells, increased ROS levels resulting in mitochondrial dysfunction, while minimally affecting healthy cells. This behavior was related to the high stability of this complex as observed in EPR investigations [72]. Also, the toxicity of several Cu(II)-coordinated carbosilane dendrimers was examined against selected tumor cell lines. It was found that the most promising compound that exhibited significant prostate tumor size reduction in a xenograft mice model and long-term survival of the animals with no signs of toxicity was the one with the higher stability which, although it showed the lowest in vitro toxicity, it exhibited the higher selectivity against cancer cells [73]. In addition, the hydrophilic–hydrophobic balance of these metallodendrimers, was also considered of importance in inducing remarkable cytotoxicity against cancer cells [74]. Pyridine-functionalized poly(amidoamine) dendrimers complexed with Cu(II) through both the internal tertiary amines and the surface pyridine groups were able to inhibit the proliferation of different cancer cell lines with low IC_50_ values and to induce significant cancer cell apoptosis [75]. Studies on metallophosphorus dendrimers prepared by the complexation of phosphorus dendrimers bearing iminopyridino end-groups as ligands of Cu(II), revealed that the uncomplexed dendrimers activated caspase-3, which plays a central role in the execution-phase of cell apoptosis, whereas the complexes promoted the translocation of the apoptosis regulator protein BAX to the mitochondria [76,77]. Our results are in line with the above reports, as although both uncomplexed PEI and PEI:Cu polymeric complexes induce apoptosis in PC3 and DU145 cells, it is clear from our cytotoxicity, mitochondrial ROS formation, and the potential measurements of the mitochondrial membrane that PEI has a different toxicity profile against these two cell lines than PEI:Cu complexes.

This work clearly shows that even simple dendritic macromolecules that have the ability to form stable copper complexes, without the need for any additional functionalization, can target specific cancerous cells, highlighting the potential of this approach despite its simplicity, and encouraging further research on this topic. Although it is not yet fully understood why these copper-coordinated compounds specifically target certain prostate cancerous cells, we can tentatively attribute our findings to the efficient cell membrane penetration of PEI:Cu derivatives, due to their reduced and more compact size, and to the fact that prostate cancer cells express high levels of the primary copper transporter CTR1 and of chaperones whose function is maintaining intracellular copper homeostasis [10]. Certainly, a more detailed study is necessary to shed light on these findings and further exploit their potential as anticancer agents.

## 4. Materials and Methods

### 4.1. Chemicals and Reagents

Hyperbranched polyethyleneimine (PEI, CAS number 9002-98-6) of 25 kDa average molecular weight (Lupasol^®^ WF, 99%) and of 750 kDa average molecular weight (Lupasol^®^ P, 50% water solution, CAS 9002-98-6) was provided by BASF (Ludwigshafen, Germany). Inverse gated ^13^C NMR analysis revealed for PEI 25 kDa that the ratio of primary to secondary to tertiary amines is 1.00:1.18:1.01 and the branching degree is 0.68, while for PEI 750ka, the ratio of primary to secondary to tertiary amines is 1.00:1.19:1.08, and the branching degree is 0.69.

CuCl_2_·2H_2_O was purchased from Sigma-Aldrich (Poole, UK). RPMI 1640 (with L-glutamine) was purchased from Biowest (Nuaillé, France), whereas phosphate buffer saline (PBS), fetal bovine serum (FBS), penicillin/streptomycin, and trypsin/EDTA were purchased from BIOCHROM (Berlin, Germany). Opti-MEM without phenol red (Gibco) was purchased from ThermoFisher Scientific (San Jose, CA, USA). Thiazolyl blue tetrazolium bromide (MTT, CAS 298-93-1) was purchased from Sigma-Aldrich Ltd. (Poole, UK). Tetramethyl rhodamine methyl ester (TMRM) and MitoSOX™ Red were obtained from ThermoFisher Scientific (Waltham, VA, USA). FITC Annexin V Apoptosis Detection Kit with 7-AAD was purchased from BioLegend (San Diego, CA, USA). High-purity dimethyl sulfoxide (DMSO) was obtained from Merck KGaA (Calbiochem^®^, Darmstadt, Germany).

### 4.2. PEI–Copper(II) Ions (PEI:Cu) Complex Formation and Characterization

For the formation of the series of PEI:Cu complexes of various Cu:N molar ratios, appropriate quantities of CuCl_2_ aqueous solution (20 mg/mL) were gradually added under rigorous stirring to a 2 mg/mL PEI solution in phosphate buffer (PB, pH = 7.4, 100 mM). The resulting solutions were allowed for 24 h at room temperature to ensure that complete equilibrium was reached. UV–Vis spectroscopy (Cary 100 Conc UV–Visible spectrophotometer, Varian Inc., Mulgrave, VIC, Australia) was employed to verify the complex formation. The corresponding spectra were registered at the wavelength range of 300–800 nm and revealed for all complexes a maximum of absorption at 632 nm. The surface charge of the parent PEI and PEI:Cu metallopolymers was determined at 25 °C using a Zetasizer Nano ZS apparatus (Malvern Instruments Ltd., Worcestershire, UK). For these experiments, aqueous solutions (5 mg/mL, pH = 7.4) of PEI (average mol. weight 750 kDa) and of the corresponding PEI:Cu polymers were employed. For each solution, ten measurements were acquired, and the results were averaged. The dynamic light scattering measurements of various concentrations of the above solutions were performed on an ALV/CGS-3 Compact Goniometer System (ALV GmbH, Langen, Germany) using a JDS Uniphase 22 mW He Ne laser operating at 632.8 nm, an Avalanche photodiode detector at an angle of 90° interfaced with an ALV-5000/EPP multi-tau digital correlator with 288 channels, and an ALV/LSE-5003 light scattering electronics unit for stepper motor drive and limit switch control. For each dispersion, at least three light scattering measurements were acquired, and the autocorrelation functions were analyzed using the CONTIN algorithm to obtain the apparent hydrodynamic radii distribution. Fits to the correlation functions were made using the software provided by the manufacturer. Before either zeta-potential or DLS measurements, all sample solutions were filtered through 0.22 μm nylon syringe filters (Membrane Solutions, Auburn, WA, USA) in order to remove any dust particles. FTIR spectroscopy was also employed for establishing the formation of PEI:Cu complexes. Since the phosphate ions would interfere in the IR spectra of the complexes, the starting PEI solution was brought to pH 7.4 by adding HCl (0.5 N), and then allowed to interact with the appropriate quantities of CuCl_2_ solution following the above procedure. The resulting solutions were lyophilized to obtain PEI:Cu complexes in the form of thick pastes, that were subsequently used for registering their FTIR spectra employing a Nicolet 6700 spectrometer (Thermo Scientific, Waltham, MA, USA) with a Specac Quest ATR diamond accessory (Specac Ltd. Orpington, Kent, UK). Spectra were recorded by averaging 64 individual scans at a resolution of 4 cm^−1^ resolution.

### 4.3. Cell Culture

The human prostate androgen-sensitive LNCaP and the androgen-insensitive cell lines with moderate DU145 and high PC3 metastatic capacity, as well as the non-cancerous human embryonic kidney HEK293 cell line used in this study, were obtained from the cell bank of the Institute of Nanoscience and Nanotechnology, NCSR Demokritos. The cell lines were free of mycoplasma contamination, as judged by regular 4′,6′-diamidino-2-phenylindole (DAPI) staining and were not used for more than 15 passages after resuscitation. Cell lines were grown in RPMI 1640 medium with stable L-glutamine, supplemented with 10% FBS and 1% penicillin/streptomycin at 37 °C in a 5% CO_2_ humidified atmosphere, and sub-cultured, twice a week, after detaching with a solution containing 0.05% (*w*/*v*) trypsin and 0.02% (*w*/*v*) EDTA.

### 4.4. Cell Viability Assay

The cytotoxicity of PEI and PEI:Cu complexes against PC3, DU145, LNCaP, and HEK293 cell lines was assessed employing the MTT assay. Cells were seeded in 96-well plates (3 × 10^3^ cells per well in 100 μL culture medium) at 37 °C in a 5% CO_2_ incubator. Cells were treated with different doses of compounds (5, 10, 25, 50, 100, and 200 μg/mL) at 37 °C for 3 h in Opti-MEM medium supplemented with 1% penicillin/streptomycin. This medium was used because it was observed that these complexes form turbid solutions in RPMI + 10% FBS medium during incubation at 37 °C when the highest concentrations were used. In addition to the control, for comparison purposes, we also treated cells with CuCl_2_ solutions at the concentration range of 9–360 μΜ, which corresponds to the Cu(II) content of the PEI:Cu 1:4 derivative at its studied concentration range (i.e., 5–200 μg/mL).

Then, the cells were washed with PBS and RPMI 1640 medium with stable glutamine supplemented with 10% FBS and 1% penicillin/streptomycin was added in each well and further incubated for the next 24 h at 37 °C in a 5% CO_2_ humidified atmosphere. Then, the mitochondrial redox function (translated as cell viability) of all cell groups was measured by the MTT assay. In brief, cell media were replaced with complete media containing MTT (1 mg/mL) and incubated at 37 °C in a 5% CO_2_ humidified atmosphere for 4 h. The 4 h incubation was followed by the aspiration of the solution and the resulted formazan crystals were solubilized in DMSO (100 μL per well) with shaking for 10 min at 100 rpm in an orbital shaker. The absorbance was measured with an Infinite M200 plate reader (Tecan Group Ltd., Männedorf, Switzerland) at a wavelength of 540 nm. The relative cell viability was calculated as cell survival percentage compared to cells that were treated only with complete medium (control). Blank values measured in wells with DMSO and no cells were in all cases subtracted. The results were expressed as % cell viability = (mean optical density (OD) of treated cells/mean OD of untreated cells) × 100. Six replicates were performed for each concentration, and the experiments were repeated in triplicate.

### 4.5. Cytofluorometric Measurements of Mitochondrial Reactive Oxygen Species (ROS) Formation and of Mitochondrial Membrane Potential

The fluorogenic dye MitoSOX™ Red was used to detect mitochondrial reactive oxygen species (ROS) formation, especially superoxide ions which are the predominant ROS in mitochondria [78,79], in live PC3, DU145, LNCaP, and HEK293 cells. Cells were seeded in 96-well black plates at a density of 10 × 10^3^ cells per well and allowed to grow overnight. Then, the cells were treated with 5 μg/mL of PEI or PEI:Cu complexes for 3 h in Opti-MEM medium supplemented with 1% penicillin/streptomycin. For comparison purposes, cells were also treated with free Cu(II) ions at a concentration of 9 μΜ that corresponds to the nominal Cu concentration in a solution of 5 μg/mL PEI:Cu 1:4 complex. Following this period, cells were washed gently with warm PBS buffer and further incubated with 5 μΜ MitoSOX™ Red at 37 °C for 20 min in the dark. The fluorescence intensity of MitoSOX™ Red was recorded by using an Infinite M200 plate reader at an emission wavelength of 580 nm and at an excitation wavelength of 510 nm.

Changes in the mitochondrial membrane potential of live PC3, DU145, LNCaP, and HEK293 cells were monitored using TMRM. The experimental procedure utilized was the same as described above for the mitochondrial ROS formation experiment. After the incubation period, TMRM was added to a final concentration of 200 nM for 20 min, and the fluorescence intensity of TMRM was measured in an Infinite M200 plate reader at an emission wavelength of 573 nm and at an excitation wavelength of 548 nm.

### 4.6. Apoptosis/Necrosis Assay

The apoptosis/necrosis assay was performed to determine the percentage of cells undergoing cellular apoptosis or necrosis induced by PEI:Cu complexes. The necrosis/apoptosis ratio of PC3 and HEK293 cells upon treatment with PEI or PEI:Cu complexes (5 μg/mL) was determined using Annexin V-FITC/7-Aminoactinomycin D (7-AAD) double staining by means of flow cytometry. Briefly, cells were grown in 6-well plates overnight, and were then treated with PEI and PEI:Cu 1:4 complexes for 3 h in Opti-MEM medium supplemented with 1% penicillin/streptomycin. Following treatment, cells were washed with PBS and incubated for 24 h in RPMI 1640 medium with stable glutamine supplemented with 10% FBS and 1% penicillin/streptomycin, at 37 °C in 5% CO_2_ humidified atmosphere. After incubation, the cells were trypsinized, suspended in PBS buffer, and centrifuged at 1000 rpm for 5 min. The cell pellet was re-suspended in 100 μL of staining buffer and 5 μL of Annexin V-FITC and 5 μL 7-AAD solution were added. Following 15 min incubation at 37 °C in the dark, the cells were analyzed with a FACSCalibur flow cytometer (Becton Dickinson, Heidelberg, Germany).

### 4.7. Statistical Analysis

At least three independent repetitions for each experiment were performed, and the data are presented as mean ± standard deviation. A Student’s *t*-test was employed to assess statistical significance for all treatments (* *p* < 0.05, ** *p* < 0.01, *** *p* < 0.001, **** *p* < 0.0001).

## Data Availability

Data are contained within the article.

## Supplementary Materials

The following supporting information can be downloaded at https://www.mdpi.com/article/10.3390/ph18081189/s1, Figure S1: (a) Zeta-potential values and hydrodynamic radii distributions of PEI of 750 KDa molecular weight and of the corresponding PEI750:Cu complexes (pH 7.4, polymer concentration 5 mg/mL); Figure S2: Annexin V-FTIC and 7-AAD staining of HEK293 cells; Figure S3: Annexin V-FTIC and 7-AAD staining of PC3 cells.

## Abbreviations

The following abbreviations are used in this manuscript:

7-AAD	7-aminoactinomycin
ATR	Attenuated total reflection
DMSO	Dimethyl sulfoxide
DLS	Dynamic light scattering
FITC	Fluorescein isothiocyanate
FTIR	Fourier transform infrared spectroscopy
MTT	Thiazolyl blue tetrazolium bromide
NMR	Nuclear magnetic resonance
ROS	Reactive oxygen species
PEI	Hyperbranched polyethyleneimine
TMRM	Tetramethyl rhodamine methyl ester
UV-vis	Ultraviolet–visible spectroscopy
