# Supplementary material for: Hyperbranched Polyethyleneimine–Coordinated Copper(II) Metallopolymers with Preferential Targeting to Prostate Cancer Cells
Source: Pharmaceuticals (Basel). 2025 Aug 12;18(8):1189. doi: 10.3390/ph18081189 (PMC12389146; doi:10.3390/ph18081189)
Supplement: Supplementary file 1 [file pharmaceuticals-18-01189-s001.zip › pharmaceuticals-3787031-supplementary.pdf]

# Hyperbranched Polyethyleneimine-Coordinated Copper(II) metallopolymers with preferential targeting to prostate cancer cells

**Barbara Mavroidi [1], Kyriaki-Marina Lyra [1], Stergios Pispas [2], Zili Sideratoy [1], and Dimitris Tsiourvas [1,*]**

Supplementary Materials

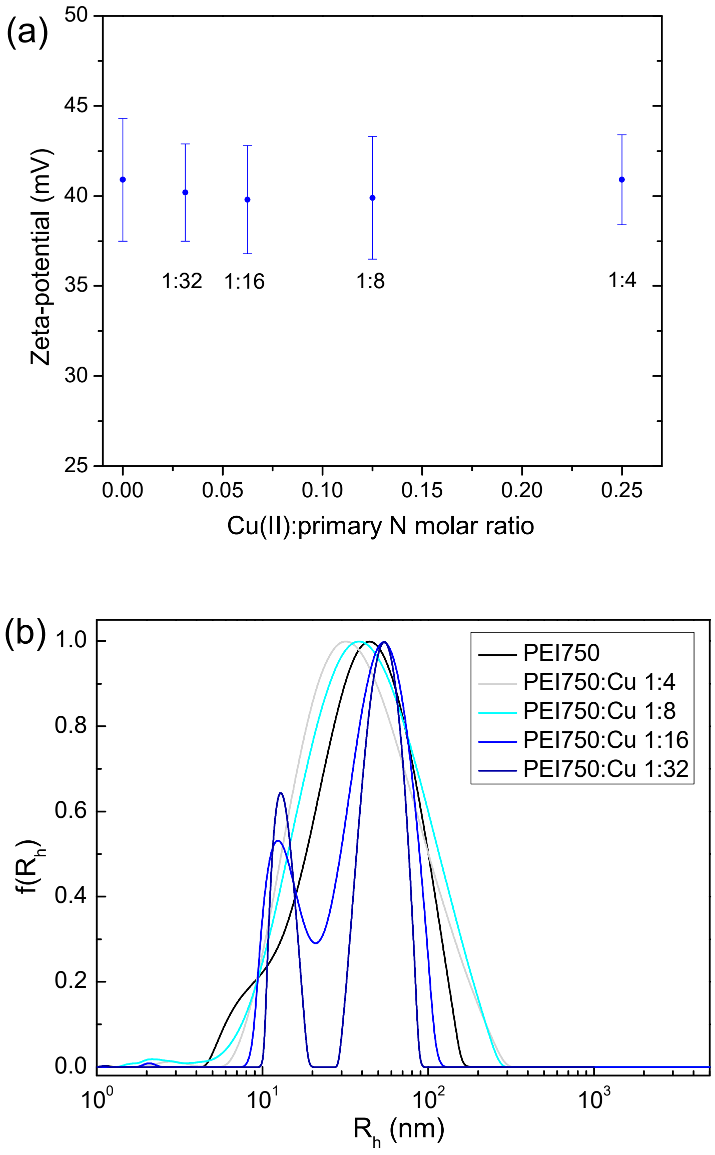


**Figure S1.** (a) Zeta-potential values of PEI of 750KDa molecular weight (PEI750) and of the corresponding PEI750:Cu complexes of various Cu(II):primary amino groups of PEI molar ratios (pH 7.4, polymer concentration 5 mg/mL) as a function of Cu(II):primary N molar ratio. Data are expressed as mean ± SD of at least 10 independent measurements; (b) Intensity weighted hydrodynamic radii distributions of PEI750 and PEI750:Cu complex solutions (pH 7.4) at the same as above concentration. Data shown are the mean of at least three dynamic light scattering measurements.

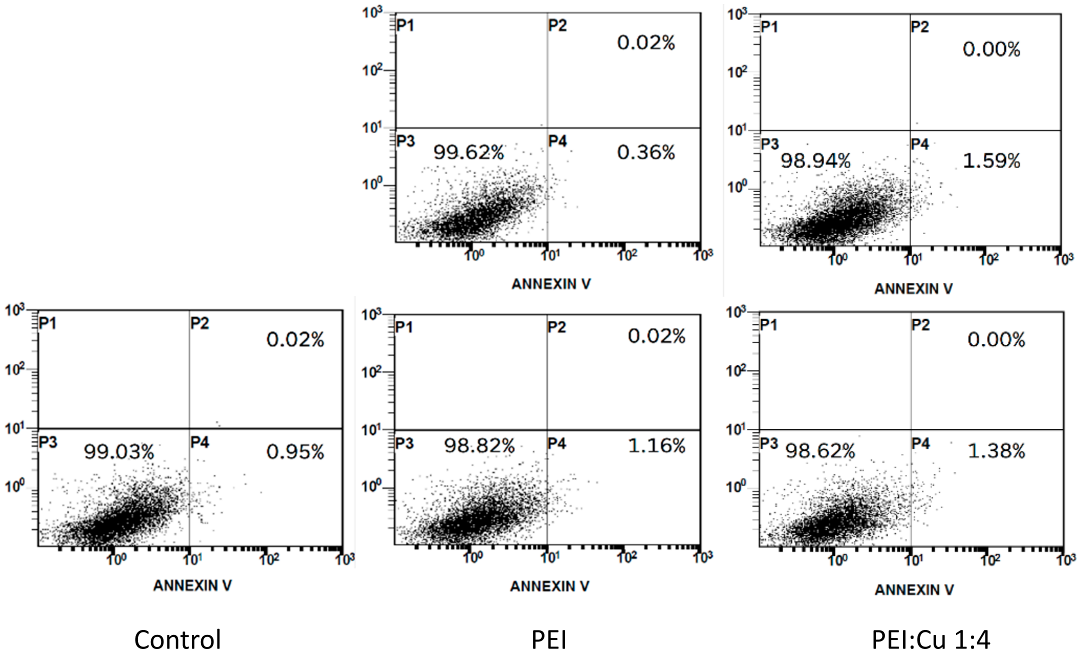


**Figure S2.** Annexin V-FTIC and 7-AAD staining of HEK293 cells: Representative flow cytometry dot plots of cells after treatment with PEI or PEI:Cu 1:4 complex (5 µg/mL, 3 h), following further incubation for 24 h. Untreated cells (Control, left panel) and treated cells (center and right panel) are shown. X and Y axes represent green (FL1-H) and red (FL3-H) fluorescence channels showing Annexin V-FTIC and 7-AAD staining, respectively. Cells were gated according to untreated cells for analyses. Each plot represents cells positive for Annexin V only (Low Right quadrant representing early apoptotic), positive for 7-AAD only (Upper Left quadrant representing necrotic or end-stage apoptotic), positive for both markers (Upper Right quadrant representing late apoptotic), and negative for both (Lower Left quadrant representing live cells). Experiments were performed as independent duplicates. Cells were stained according to conventional Annexin V/7-AAD protocol.

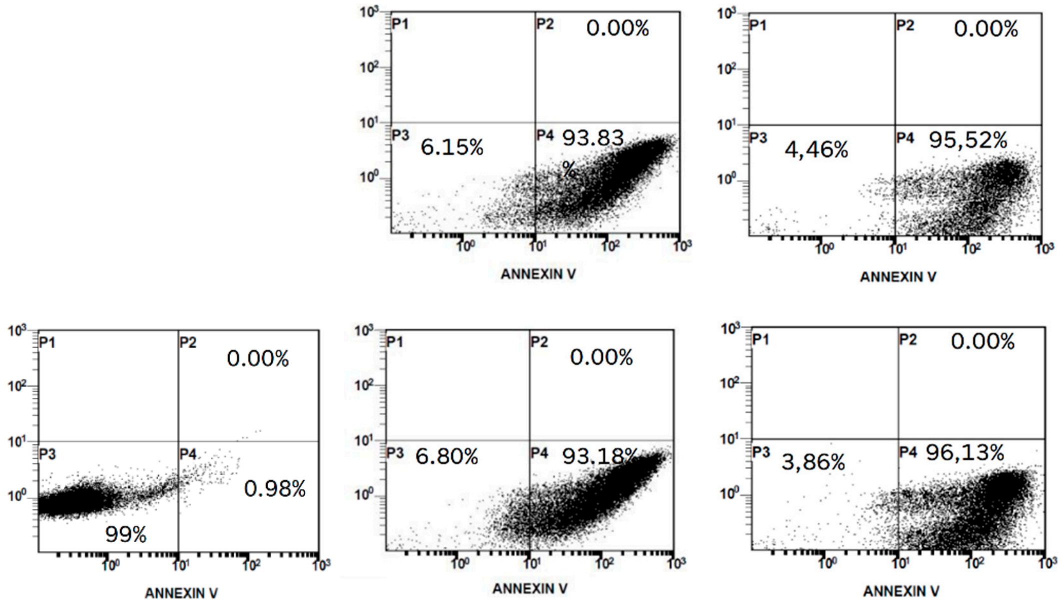


Control PEI PEI:Cu 1:4

**Figure S3.** Annexin V-FTIC and 7-AAD staining of PC3 cells: Representative flow cytometry dot plots of cells after treatment with PEI or PEI:Cu 1:4 complex (5 µg/mL, 3 h), following further incubation for 24 h. Untreated cells (Control, left panel) and treated cells (center and right panel) are shown. X and Y axes represent green (FL1-H) and red (FL3-H) fluorescence channels showing Annexin V-FTIC and 7-AAD staining, respectively. Cells were gated according to untreated cells for analyses. Each plot represents cells positive for Annexin V only (Low Right quadrant representing early apoptotic), positive for 7-AAD only (Upper Left quadrant representing necrotic or end-stage apoptotic), positive for both markers (Upper Right quadrant representing late apoptotic), and negative for both (Lower Left quadrant representing live cells). Experiments were performed as independent duplicates. Cells were stained according to conventional Annexin V/7-AAD protocol.
